# Museomics unravels cryptic diversity in an endemic group of New Guinean songbirds

**DOI:** 10.1098/rsbl.2024.0611

**Published:** 2025-07-16

**Authors:** Mozes Pil Kyu Blom, Saphira Bloom-Quinn, Petter Zahl Marki, Bonny Koane, Leo Joseph, Martin Irestedt, Knud Andreas Jønsson

**Affiliations:** ^1^ Museum für Naturkunde - Leibniz-Institute for Evolution and Biodiversity Science, Berlin 10115, Germany; ^2^ University of Copenhagen Natural History Museum of Denmark, Kobenhavn, Region Hovedstaden, Denmark; ^3^ Division of Research & Innovation, University of Agder, Kristiansand, Vest-Agder, Norway; ^4^ New Guinea Binatang Research Centre, Madang, Papua New Guinea; ^5^ Australian National Wildlife Collection, CSIRO, Canberra, Australian Capital Territory, Australia; ^6^ Department of Bioinformatics and Genetics, Swedish Museum of Natural History, Stockholm, Sweden

**Keywords:** museomics, phylogenomics, speciation, biodiversity, taxonomy, jewel-babblers

## Abstract

Deciphering cryptic diversity can have substantial implications for our understanding of evolutionary processes and species conservation. Birds are arguably among the best studied organismal groups, but even in avian clades there are some genera that have not been thoroughly surveyed. This is particularly true for taxa that occur in hyperdiverse biogeographic regions. In this study, we focus on an endemic group of New Guinean birds, the jewel-babblers (genus: *Ptilorrhoa*), and study the diversification history of all known taxa. We assemble a de novo genome using linked-read sequencing and genomic data for 40 historical specimens. Both phylogenomic and population-genomic analyses strongly support the recovery of a cryptic species and shed new light on the diversification history of this group. The blue jewel-babbler (*Ptilorrhoa caerulescens*) is a paraphyletic species complex and *P. c. nigricrissus* is more closely related to the phenotypically distinct and sexually dimorphic *P. geislerorum*, than to other *P. caerulescens* subspecies. These findings demonstrate that even in well-studied groups such as birds, cryptic diversity can still be a prevalent reality. Moreover, by deciphering cryptic diversity, we shed new light on the processes driving speciation within *Ptilorrhoa* and the need to potentially revise the taxonomic status of all subspecies.

## 1. Introduction

To study the processes that promote and sustain biodiversity, accurately cataloguing species and population diversity across space and through time is a key prerequisite. This is particularly important today as anthropogenic alterations of the planet have led to a biodiversity crisis, in which the extinction rate is estimated to have accelerated up to 100 times [1,2]. Apart from direct habitat loss, e.g. anthropogenic deforestation, it is predicted that global warming will have the largest effects on biodiversity in the tropics [3], where local extinctions due to climate change have already been demonstrated [4]. Moreover, a significant proportion of species that go extinct may not even be described by science, so-called ‘dark extinctions’ [5]. Improved knowledge about biodiversity and the processes that have shaped it is therefore key for a full understanding of the anthropogenic biodiversity crisis and which measures may be most effective to curb the ongoing mass extinction.

New Guinea is the world’s largest tropical island and one of the most diverse places on Earth. It is estimated that New Guinea hosts 10–20% of terrestrial biodiversity in an area that constitutes less than 0.5% of Earth’s land area [6]. New Guinea has a complex geological history [7–9] and is today dominated by a long, high central mountain range that separates the island’s northern lowlands from the southern lowlands, the latter of which have been connected to Australia during times of low sea-levels [9]. Scattered throughout the lowlands are smaller mountain areas that can be characterized as montane sky-islands. With its steep mountains and expansive lowlands as well as a varied and complex climatic and geological history, New Guinea represents a microcosm, ideal to explore how biodiversity has built up over time and how it is distributed across the landscape. The island has consequently been important for the development of evolutionary and ecological theories [10–13].

While the evolutionary and biogeographic history of New Guinean flora and fauna has been of longstanding interest, few studies have attempted to characterize phylogeographic diversity across the island using genome-scale data. For example, recent efforts to determine how the avifauna has formed and attained its current distributions were limited to species level sampling (e.g. [12]) or focused on select population comparisons [13,14]. While studies with more dense sampling have mostly relied on single mitochondrial genes [15–18]. However, limited sampling, across the landscape or genome, can hamper our ability to characterize phylogeographic and macroevolutionary diversity, and as a consequence bias our capacity to study the mechanisms that shape biodiversity. A primary reason for the paucity of such genomic studies is the underlying challenge of gathering a representative sample of phylogeographic diversity across the heterogeneous landscape, which includes many remote regions and difficult to reach mountain ranges. To address this issue, we have turned to natural history collections and complement contemporary samples with historical specimens to study the diversification history of an endemic group of New Guinean birds, the jewel-babblers (genus: *Ptilorrhoa*).

We used a linked-read sequencing approach to assemble a draft genome for the spotted jewel-babbler (*Ptilorrhoa leucosticta*) and generated whole-genome resequence data for all four species (including all 17 recognized subspecies) and an outgroup (genus: *Cinclosoma*). Our phylogeographic and genome-wide sampling provides a robust phylogenetic and phylogeographic framework to properly assess taxonomic delimitations of jewel-babblers and reveals a surprising case of cryptic diversity. Altogether, these findings highlight the value of integrating natural history collections and genomics in biodiversity research and how such approaches can provide insights into the processes shaping biodiversity.

## 2. Methods

### (a) Specimen sampling and sequencing

The endemic jewel-babblers of New Guinea (genus: *Ptilorrhoa*) comprise 17 named taxa divided into four recognized species. Together with quail-thrushes (genus: *Cinclosoma*) they represent one family (Cinclosomatidae) out of 31 Corvides families within passerine birds. The taxonomic division is based on plumage patterns and geographic distributions, which span the lowlands (*Ptilorrhoa caerulescens* (0−300 m.a.s.l.) and *P. geislerorum* (0−1200 m.a.s.l.)), lower montane elevations (*P. castanonota* (900–1450 m.a.s.l.)) and the highlands (*P. leucosticta* (1750–2400 m.a.s.l.)). Thus, *Ptilorrhoa* species represent various examples of geographical and elevational displacement across New Guinea. To cover the biogeographic distribution of the region, we complemented tissue samples of nine contemporary samples with 32 toepad samples from historical specimens (incl. a single *Cinclosoma punctatum dovei* individual to be used as outgroup: electronic supplementary material, table S1). For DNA extractions and genome library preparation from fresh and historical tissue samples, see [19] and electronic supplementary material. Whole-genome resequencing data were generated on Illumina platforms (HiSeq2500 and HiSeq X) by SciLifeLab, Stockholm. In addition to the short-read resequencing data, we used a frozen fresh tissue sample of *Ptilorrhoa leucosticta loriae* to extract high-quality DNA. A linked-read sequencing library was then generated using the 10× Chromium platform and sequenced on an Illumina HiSeq X by SciLifeLab, Stockholm.

### (b) Bioinformatic processing and de novo assembly

Both the short- and linked-read sequencing data were processed with a suite of custom-designed Nextflow (nf) pipelines (see electronic supplementary material for more detail). We first used Nf-core Neutronstar [20] for a de novo assembly of the *Ptilorrhoa leucosticta loriae* reference genome. Nf-core Neutronstar uses Supernova (v 2.1.1 [21]) for initial assembly, followed by QUAST (v. 5.0.2 [22]) and BUSCO (v. 3.0.2 [23]) to evaluate assembly statistics. We then used RagTag (v. 2.1.0 [24]) Scaffold to compare the 10× Chromium scaffolds with the empirically informed chromosome-scale reference genome of *Lycocorax obiensis* [25] and placed the 79 longest scaffolds (representing 90% of the genome) on their corresponding chromosomes. With a reference genome in place, we used nf-polish (https://github.com/MozesBlom/nf-polish) to clean raw sequencing reads and nf-umap (https://github.com/IoMue/nf-umap) to map the polished reads to the *P. l. loriae* reference. We then repeated the same workflows, but mapped the polished reads to a previously published mitochondrial genome of *P. leucosticta* (GenBank: MN356166.1 [26]).

### (c) Population structure

We used ANGSD (v. 0.9.2 [27]) to call genotype likelihoods (GL) and characterize inter- and intraspecific species structure. GL calls allow us to take coverage differences into account and are particularly suitable when including ancient or historical specimens. In this study, we use both contemporary and historical specimens and first wanted to account for any possible biases associated with the use of museum specimens. In example, to investigate whether individuals would cluster by tissue type rather than species. We called GL’s for all *Ptilorrhoa* individuals and linkage pruned the resulting datasets by only retaining every 20th or 50th call. We then conducted a principal component analysis (PCA) with PCAngsd (v.1.21 [28]) and inferred admixture proportions with NGSadmix (v0.935 [29]).

### (d) Phylogenetic analyses

We used nf-var (https://github.com/MozesBlom/nf-var) to call and filter variants for all individuals, generate Mask files and consensus sequences per chromosome (see electronic supplementary material). The Mask file lists sites that did not fulfil filtering criteria and the consensus sequence includes 'Ns’ at the corresponding positions. We ran nf-var twice, first to generate consensus sequences for the mitochondrial genome and second to call consensus sequences for the nuclear genome. We aligned all mitochondrial genomes and used IQ-TREE 2 (v. 2.2.2.6 [30]) to infer a maximum-likelihood species tree.

Our primary aim for anchoring our 10× chromium scaffolds to a chromosome scale reference was to differentiate between autosomes and sex chromosomes for the phylogenetic analyses. We used nf-phylo (https://github.com/MozesBlom/nf-phylo/) to align our consensus sequences by chromosome, subset each chromosome into genomic windows 10 kb in length sampled at a minimal distance of 100 kb and reconstruct species trees. Each genomic window was filtered for missing data and we used IQtree2 (v. 2.2.2.6 [30]) to infer a maximum-likelihood (ML) species tree for each genomic window. Moreover, we combined all filtered genomic windows per chromosome and across all autosomes, to infer ML species trees based on concatenated alignments. Then, we parsed all genomic window trees and used ASTRAL-III [31] to infer a summary-coalescent species tree for all *Ptilorrhoa* and rooted the species tree at the *Cinclosoma p. dovei* outgroup. Using both alignments and genomic window trees, we calculated both site- and window-concordance factors [32] for all species trees.

While nf-phylo provides a genome-scale estimate of the species-tree, it is computationally intractable to use the same dataset for divergence dating. We therefore randomly sampled 1000 of the 10 kb autosomal windows and repeated this five times. Since these window alignments were already quality filtered with nf-phylo, all datasets were highly complete with most individuals (*n* = 38) having less than 3% missing data across all five subsets (electronic supplementary material, table S2). We concatenated all window alignments for each of the five subsets and used BEAST2 (v. 2.7.7 [33]) to scale branch lengths with a root age prior. Without any dated fossils available for this genus, we used the mean estimate (16.1864 My) and the 95% HPD distribution (10.8484−21.4499) for the split between *Cinclosoma* and *Ptilorrhoa* as inferred by Oliveros *et al.* [34]. They used ultra-conserved elements and more than 10 fossils to infer and date the entire passerine radiation. We ran BEAST2 for 10 million generations, after which convergence was reached for each of the five datasets. The five randomly sampled datasets resulted in virtually identical phylogenies and only marginally varied in terms of divergence time estimates.

Finally, given the observed paraphyly of *P. caerulescens,* we compared the relative frequencies of competing four-taxon gene tree topologies to test for putative introgressive hybridization between *P. caerulescens* subspecies [35]. We used nf-GT (https://github.com/MozesBlom/nf-GT/) to estimate the topological frequencies of quartet trees (for convenience referred to ABBA, BABA and BBAA topologies) for each Cartesian combination of individuals between *Cinclosoma, P. c. caerulescens/neumanni, P. c. nigricrissus* and *P. geislerorum*. Similar to Patterson’s D [36], under a null hypothesis of ILS only, the two competing topologies (ABBA and BABA) should be recovered in equal numbers whereas a skew in ABBA or BABA topologies would be indicative of (ancestral) hybridization between *P. c. caerulescens/neumanni* and *P. c. nigricrissus* or *P. geislerorum*. More detail on all bioinformatic processing steps and analyses can be found in the electronic supplementary material.

## 3. Results and discussion

**Figure 1.**
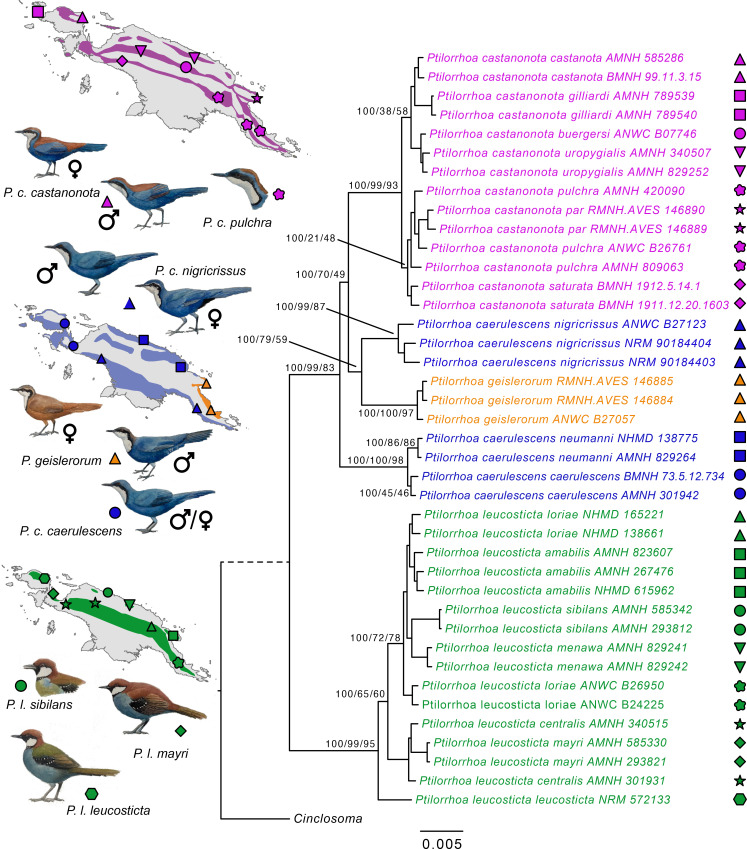
Phylogenetic history of *Ptilorrhoa* species with biogeographic distributions. The phylogeny was inferred using maximum-likelihood and a concatenated dataset of 10 kb autosomal genomic windows sampled at 100 kb intervals. The phylogeny has been manually rooted at the *Cinclosoma p. dovei* outgroup. Numbers at nodes indicate bootstrap support values, window- and site-concordance factors. Population sampling sites are indicated on the geographic map of New Guinea. Illustrations of *Ptilorrhoa* species (by Jon Fjeldså) are presented with an emphasis on plumage differences between sexes and across species. Tip names are coloured to indicate currently recognized species. The branch leading to the outgroup *Cinclosoma p. dovei* is dotted to indicate that the branch has been shortened for graphical purposes.

**Figure 2.**
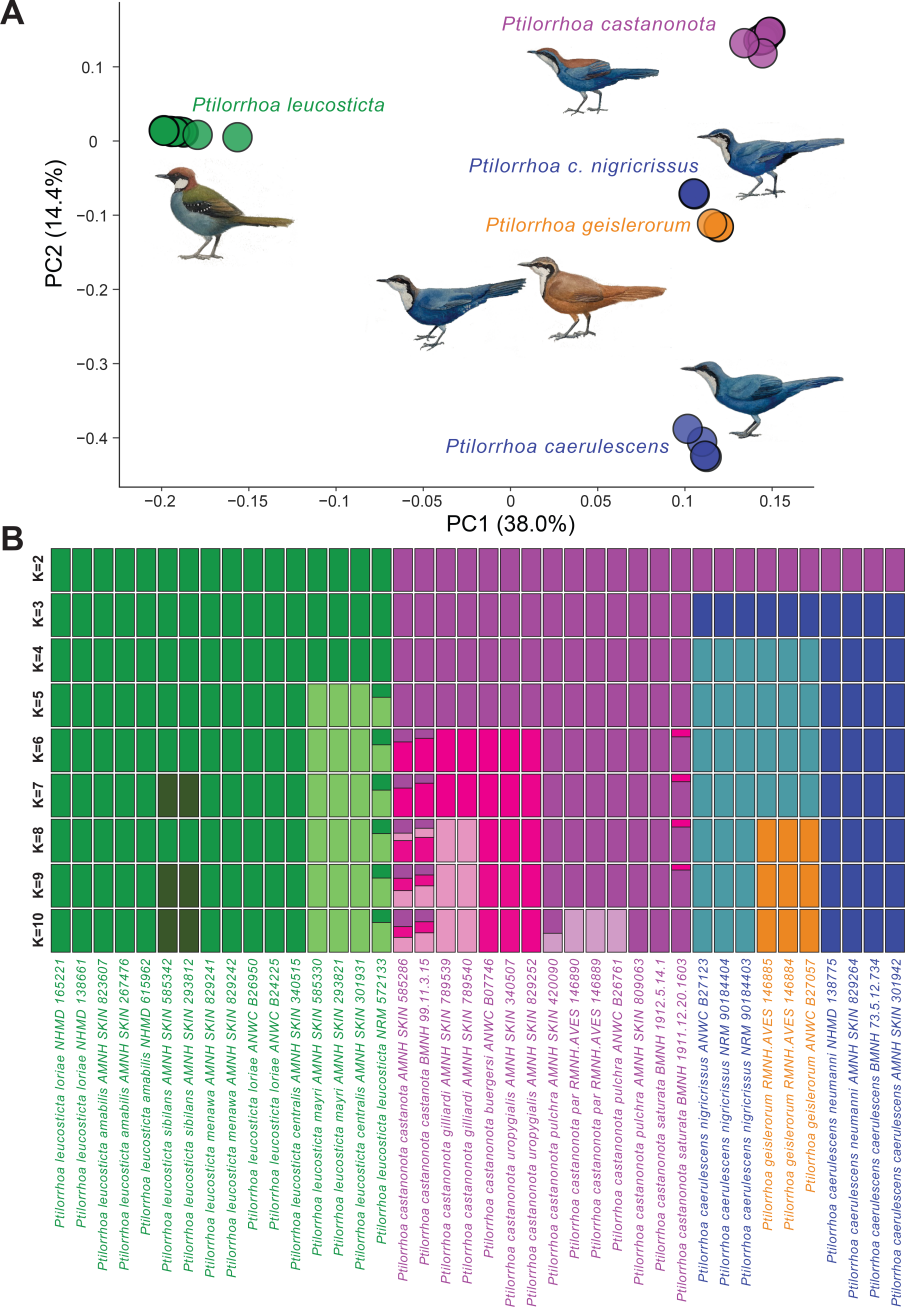
Population structure and genome composition. (A) Principal Component Analyses and (B) admixture analyses based on genotype likelihoods. *Ptilorrhoa caerulescens nigricrissus* is more similar to *P. geislerorum* than to other *Ptilorrhoa caerulescens* subspecies.

Phylogeographic sampling of the entire *Ptilorrhoa* genus (including data for all currently recognized species (*n* = 4) and subspecies (*n* = 17)) and whole-genome resequencing, provide an unprecedented and surprising view on species diversity and biogeographic history of an endemic New Guinean avian radiation. Even in a relatively well-studied group such as songbirds, our genomic characterization provides unanimous support for a surprising case of cryptic diversity. Species trees based on 8097 autosomal or 614 Z-linked loci (figure 1, electronic supplementary material, figures S1–S2), each 10 kb in length, or mitochondrial genomes (electronic supplementary material, figure S3), all suggest that the blue jewel-babbler (*P. caerulescens*) comprises taxa that are paraphyletic with respect to each other. *P. c. nigricrissus* is more closely related to the dimorphic jewel-babbler (*P. geislerorum*) than to other blue jewel-babbler taxa. Moreover *P. c. nigricrissus/P. geislerorum* form a clade that is sister to the chestnut-backed jewel-babblers (*P. castanonota*) rather than to the other blue jewel-babblers. Similarly, analyses based on covariance between allele frequencies across 1 014 010 loci also indicate a paraphyletic status of the blue jewel-babbler, with the three *P. c. nigricrissus* individuals clustering with *P. geislerorum* in both the PCA and admixture analyses (figure 2, electronic supplementary material, figure S4).

Cryptic diversity as observed here can be the outcome of phenotypic stasis/convergence [37] or due to processes that lead to deep coalescence (e.g. incomplete lineage sorting, ILS [38] or hybridization [39]). However, by taking a genome-wide approach, we find strong support that the latter is not causing the paraphyletic patterns observed for the blue jewel-babblers. The summary-coalescent species tree (electronic supplementary material, figure S1), which explicitly accounts for ILS, is congruent with the phylogeny based on concatenation of the same loci (figure 1) and the concordance factors further underline that a substantial majority of gene trees support a paraphyletic hypothesis. Secondly, with a lower effective population size, ILS should be less pronounced on the Z chromosome than the autosomes ([40]; electronic supplementary material, figure S2). The phylogenies based on concatenated loci are identical between both types of chromosomes and, in line with faster lineage sorting on Z, a larger proportion of gene trees support a monophyly of *P. c. nigricrissus/P. geislerorum/P. castanonota* relative to the *P. c. caerulescens P. c. neumanni* clade (92 versus 69.8; electronic supplementary material, figure S2 and figure 1). Similarly, the high degree of concordance (both site and window) across the genome also diminishes the likelihood that hybridization has led to paraphyletic relationships. In theory, ancestral hybridization between *P. c. nigricrissus* and *P. geislerorum* could have led to an overwhelming degree of introgression on both the autosomes and Z-chromosome, and this could have been to such an extent that the coalescent signal supporting the species tree has largely been overridden. However, this is highly unlikely given the dimorphic nature of *P. geislerorum* (reducing the likelihood of hybridization due to strong sexual selection), the sedentary nature of jewel-babblers and the lack of geographic overlap between *P. c. nigricrissus* and *P. geislerorum*. Lastly, the morphological similarity between *P. c. nigricrissus* and *P. c. caerulescens* could have been caused by adaptive introgression but our explicit test for introgressive hybridization rejects this hypothesis (electronic supplementary material, figure S5). Altogether, these results suggest that the current taxonomy does not reflect the true species diversity and that the blue jewel-babblers should be considered two species: *Ptilorrhoa caerulescens* (including *P. c. neumanni*) of the northern lowlands (including the Bird’s Head and Bird’s Neck of New Guinea) and *Ptilorrhoa nigricrissus* of the southern lowlands of New Guinea.

By unravelling the cryptic diversity among blue jewel-babblers, we have shed new light on the processes driving speciation within *Ptilorrhoa* and the potential need to elevate other subspecies to species rank as well. For example, in the admixture plots (figure 2), at the level of *K* (*K* = 8) where *P. c. nigricrissus* is independently recovered from *P. geislerorum,* there are also multiple distinct groups within the spotted jewel-babbler (*P. leucosticta*) and the chestnut-backed jewel-babblers (*P. castanonota*). These admixture patterns match the phylogenetic substructure within species and are consistent with a general New Guinean biogeographic pattern in which avian populations in the Bird’s Head are separated from populations on the southern slope of the Central Range, which again are separated from populations on the northern slope of the Central Range (e.g. *Ornorectes cristatus* [41] and *Melampitta* [42]). In line with their sedentary nature and low dispersal ability, these findings suggest that biogeography and population connectivity play an important role in promoting (allopatric) speciation without overt changes in phenotype. However, it is important to note that phenotype in these cases often equates to morphometric and plumage characteristics based on museum collections, since other components of phenotype (e.g. behavioural observations) often lack for populations in remote parts of New Guinea. Interestingly, the possible relevance of other phenotypic traits and observational data is further emphasized by a recent field study, which reported that parapatric blue jewel-babbler populations, including *P. c. nigricrissus,* in the Bird’s Head Peninsula show elevational displacement and may be vocally distinct [43]. Such field observations combined with the lack of plumage differentiation between various subspecies (including *P. c. nigricrissus*, *P. c. caerulescens* and, to a lesser extent, *P. geislerorum* males) suggests that sexual selection for a specific male morph may be relatively static and has not diverged despite longer periods of genetic isolation between (sub-)species. In example, males of *P. c. nigricrissus* and *P. c. caerulescens* have remained almost identical even though they split more than approximately 6 Mya (electronic supplementary material, figure S6).

This study provides further evidence that the biodiversity of tropical birds, and New Guinean biodiversity more broadly, has been consistently underestimated [41,42,44]. Our results also contribute empirical support to the idea that cryptic diversity is common in tropical environments, even within well-studied groups such as songbirds, and that a synthesis of genetic data with morphological, vocal and distributional data is required to recognize true species diversity [45]. Ergo, taxonomy solely based on morphology can be misleading, particularly in highly diverse tropical lineages, but current taxonomy and interpretations of species limits in these groups is still often based on morphological patterns. Genomic approaches should be levied to review taxonomic hypotheses and become a standard to support taxonomic decisions. In parallel, our study also emphasizes the increasing importance of natural history collections, and how museomics can be used to survey biodiversity at a population level scale [46]. Doing so may uncover unexpected instances of cryptic diversity and provide insights into the evolutionary mechanisms promoting diversification. Such efforts are increasingly urgent in understudied areas in the face of the current biodiversity crisis.

## Data Availability

All raw sequencing data have been deposited in the European Nucleotide Archive and can be publicly accessed via project number: PRJEB80871. The majority of the code used for data processing and phylogenetic analyses have been custom built in Nextflow workflows and can be accessed at: [47]. Nf-polish cleans short-read resequence data for both historical and modern specimens, nf-var was used for variant calling, filtering and consensus calling, nf-phylo was used for all phylogenetic analyses and nf-GT for introgression testing using gene trees. Each workflow comes with detailed instructions and a corresponding yaml files to recreate conda environments for reproducibility. Static versions of the workflows are available from the Dryad Digital Repository [48]. Supplementary material available online [49].
